# Consumption in San Antonio

**Published:** 1897-03

**Authors:** R. Menger

**Affiliations:** City Physician, San Antonio, Tex.


### TEXAS MEDICAL JOURNAL.

ESTABLISHED JULY, 1885.

PUBLISHED MONTHLY.—SUBSCRIPTION $2.00 A YEAR.

VOL. XII. AUSTIN, MARCH, 1897. NO. 9.

### Original Contributions.

For the Texas Medical Journal.

### CONSUMPTION IN SAN ANTONIO.

BY R. MENGER, M. D., CITY PHYSICIAN, SAN ANTONIO, TEX.

THE FOLLOWING letter, from City Physician Menger, enclosing statistics on consumption in San Antonio, will be of unusual interest at this time, when the subject of quarantining consumptives is being discussed. Dr. Menger has kept complete records of all deaths from consumption, together with age, occupation, nativity, residence, etc., of persons dying in San Antonio for several years.—ED.]

SAN ANTONIO, TEXAS, February 18, 1897.

*Editors Texas Medical Journal:*

Believing the enclosed official records of consumptives in San Antonio during the last two years will be interesting reading matter to your many readers and others, I send you same with the request to publish if deemed best by you, adding or leaving out whatever necessary. It is a striking fact, that San Antonio, with its many very old dwelling quarters and boarding houses, where cousumptives have lodged formerly, and also many ambulating cases yearly coming here seeking climatic changes, etc., and with our generally very dusty streets, and the many chances of direct infection through dried sputa-bacilli, —by all these favorable influences,—it is a puzzle to me that not more native residents become infected and die from phthisis in San Antonio. I have not heard cf one case being directly infected by phthisical nurses, either private or at the infirmaries. Dr. Herff, Sr., one of our most experienced colleagues, has had the same experience during his long practice here and in Boerne, Texas, where a very large per centage of foreign consumptives seek recuperation. It would be interesting to hear from others on this important matter, giving statistics, if possible, and actual observations, and publish same in the TEXAS MEDICAL JOURNAL.

Sincerely yours,

DR. R. MENGER.

---

MORTURARY STATISTICS—STATEMENT OF DEATHS FROM CONSUMPTION IN SAN ANTONIO DURING THE PAST TWO YEARS.

CITY HOSPITAL STATISTICS.

The following tabulated statement shows the record of the consumptive patients at the city hospital during 1895. All nonresidents:

| | Total number of all classes of diseases treated, surgical and clinical. | Total number of consumptives admitted and treated. | Deaths from all causes except consumption. | Deaths from consumption. |
|---|---|---|---|---|
| Jan | 55 | 14 | 3 | 4 |
| Feb | 70 | 7 | 1 | 3 |
| March | 65 | 10 | 5 | 2 |
| April | 56 | 5 | 1 | 3 |
| May | 52 | 7 | 0 | 1 |
| June | 54 | 5 | 2 | 2 |
| July | 56 | 9 | 0 | 2 |
| Aug | 57 | 6 | 4 | 3 |
| Sept | 53 | 4 | 4 | 1 |
| Oct | 50 | 3 | 5 | 0 |
| Nov | 46 | 5 | 2 | 2 |
| Dec | 55 | 7 | 3 | 4 |
| Total | 559 | 82 | 30 | 27 |

According to ages.—From 10 to 20, 2; 20 to 40, 40; 40 to 60, 14; 60 to 80, 2; age not stated, 24.

Occupations.—Laborers, 17; cook, 8; railroad men, 5; salesmen, 4; painters, 3; bookkeepers, 3; stone cutters, 2; clerks, 3; engineers, 2; farmers, 2; cigarmakers, 2; firemen, 2; brewers, 2; bricklayer, 1; carpenter, 1; milkman, 1; teamster, 1; drummer, 1; undertaker, 1; barber, 1.

Comparative record of city hospital in 1896. All non-residents:

| | Total number of all classes of diseases treated, surgical and clinical. | Total number of consumptives admitted and treated. | Deaths from all causes except consumption. | Deaths from consumption. |
|---|---|---|---|---|
| Jan | 49 | 8 | 3 | 4 |
| Feb | 55 | 8 | 3 | 2 |
| March | 57 | 8 | 5 | 10 |
| April | 46 | 5 | 3 | 3 |
| May | 48 | 5 | 2 | 2 |
| June | 48 | 6 | 1 | 4 |
| July | 49 | 5 | 3 | 4 |
| Aug | 39 | 1 | 3 | 4 |
| Sept | 45 | 6 | 0 | 2 |
| Oct | 63 | 8 | 0 | 1 |
| Nov | 57 | 9 | 1 | 2 |
| Dec | 69 | 14 | 3 | 0 |
| Total | 625 | 75 | 27 | 38 |

According to ages.—From 10 to 20, 5; 20 to 40, 58; 40 to 60, 19; 60 to 80, 2.

Occupations.—Laborers, 14; railroad men, 10; farmers, 8, stone cutters, 3: clerks, 3; painters, 3; miners, 3; bricklayers, 2; jewelers, 2; candy makers, 2; salesmen, 3; plasterers, 2; bakers, 2; teachers, 2; bookkeepers, 2; cooks, 2; brewer, 1; sailor, 1; book printer, 1; salesman, 1; waiter, 1; laundryman, 1; shoemaker, 1; cigar maker, 1; barber, 1; painter, 1; typesetter, 1; steel worker, 1; hotel man, 1; engineer, 1; carpenter, 1; gardener, 1; dressmaker, 1; newspaper man, 1; cowboy, 1.

As long as there are no special sanitariums under State control for consumptives, and so long as consumptives come or are promiscuously sent here from other sections, they will sooner or later fall a burden on the city. Many come or are sent here in such advanced stages of phthisis that they would surely fare better by staying near their relatives and friends instead of making the long trip to San Antonio and dying among strangers. But there can be no restriction, it seems, and hence our city is yearly burdened with a large number of phthisical patients with very limited means; and these unfortunates not being allowed to stay in the hotels and boarding houses, have to be taken care of in the city hospital at the expense of the city.

Another question that has been confronting the municipal authorities is whether consumption be a direct communicable or microbic disease. So far as northern cities are concerned, the latter view seems to be verified, the New York Board of Health having declared it to be such, and having adopted suitable precautionary and compulsory measures of prevention. As far, though, as San Antonio is concerned, our statistics rather tend to show that phthisis is almost entirely confined to patients from the outside coming here for treatment or recuperation. The following statistics compiled by me last year and approved by the board of health, corroborates this:

| Year | Estimated population. | Deaths from all causes. | Deaths from phthisis pulmonalis. | Other tubercular diseases. |
|---|---|---|---|---|
| 1885 | 35,000 | 731 | 150 | 13 |
| 1886 | 36,000 | 775 | 134 | 19 |
| 1887 | 40,000 | 880 | 118 | 30 |
| 1888 | 42,000 | 827 | 140 | 26 |
| 1889 | 46,000 | 925 | 156 | 27 |
| 1890 | 50,000 | 911 | 108 | 24 |
| 1891 | 52,000 | 1,160 | 169 | 34 |
| 1892 | 52,000 | 1,436 | 207 | 42 |
| 1893 | 54,000 | 1,098 | 169 | 21 |
| 1894 | 56,000 | 1,205 | 213 | 21 |
| 1895 | 60,000 | 1,092 | 214 | 23 |

The total number of all classes of patients in 1895 and 1896, includes all those patients transferred from one month to another, so that during the year there really were admitted for treatment in 1895, 353, and in 1896, 318 individual cases. As to consumptives, there were admitted, as stated in the tabulated report, 82 individual cases in 1895, and 75 in 1896, but the total number treated during each month, including those transferred from one month to the other, in addition to new comers, averaged about 20 to 40 per month.

It will be seen that during the eleven years the mortality from phthisis has not increased, but to the contrary, decreased, if the increase of population between 1885 and 1895 be considered. During eleven months in 1895, the total number of deaths from all tubercular diseases were 214; of those 44 were natives of Texas; 24 lived in San Antonio three and one-half years and upward, and 20 lived in San Antonio two years and less. During the eleven months of 1885, three died, out of 131 deaths from all tubercular diseases, 89 natives of Texas, or 30 per cent. In the eleven months of 1895, out of 204 deaths from all tubercular diseases 44 were Texans, or 21 per cent.; in the eleven months of 1885, 131 died, out of a population of 35,000 inhabitants, or 38-100 of 1 per cent., and in the eleven months of 1895, out of a population of 60,000 inhabitants, 204 died of tubercular diseases, or 34-100 of 1 per cent., or a decrease of 2-100 of 1 per cent. in 1895.

The city hospital is divided into four main wards, separated by large halls. The large ward on the east side of the building is used exclusively for consumptive patients, there being plenty of sunshine and ventilation. The rooms of this ward, as also of the other portions of the hospital, are scrubbed daily, and the consumptives are not allowed to spit promiscuously around the ward. They are provided with laage cuspidores, and printed instructions are posted on the walls for their guidance. The rooms are kept scrupulously clean, and are fumigated daily. In good weather as much outdoor exercise inside the hospital grounds is allowed the patients as is possible.

